# Comparable cerebral cortex activity and gait performance in elderly hypertensive and healthy individuals during dual‐task walking: A fNIRS study

**DOI:** 10.1002/brb3.3568

**Published:** 2024-07-10

**Authors:** Qiuru Yao, Ling Chen, Hang Qu, Weichao Fan, Longlong He, Gege Li, Jinjing Hu, Jihua Zou, Guozhi Huang, Qing Zeng

**Affiliations:** ^1^ Department of Rehabilitation Medicine Zhujiang Hospital, Southern Medical University Guangzhou China; ^2^ School of Nursing Southern Medical University Guangzhou China; ^3^ School of Rehabilitation Medicine Southern Medical University Guangzhou China; ^4^ Faculty of Health and Social Sciences The Hong Kong Polytechnic University Hong Kong China

**Keywords:** aging, cognition, dual‐task walking, executive functions, fNIRS, gait, hypertension

## Abstract

**Background:**

Hypertension increases the risk of cognitive impairment and related dementia, causing impaired executive function and unusual gait parameters. However, the mechanism of neural function illustrating this is unclear. Our research aimed to explore the differences of cerebral cortex activation, gait parameters, and working memory performance between healthy older adults (HA) and older hypertensive (HT) patients when performing cognitive and walking tasks.

**Method:**

A total of 36 subjects, including 12 healthy older adults and 24 older hypertensive patients were asked to perform series conditions including single cognitive task (SC), single walking task (SW), and dual‐task (DT), wearing functional near‐infrared spectroscopy (fNIRS) equipment and Intelligent Device for Energy Expenditure and Activity equipment to record cortical hemodynamic reactions and various gait parameters.

**Results:**

The left somatosensory cortex (L‐S1) and bilateral supplementary motor area (SMA) showed higher cortical activation (*p <* .05) than HA when HT performed DT. The intragroup comparison showed that HT had higher cortical activation (*p <* .05) when performing DT as SW. The cognitive performance of HT was significantly worse (*p <* .05) than HA when executing SC. The activation of the L‐S1, L‐M1, and bilateral SMA in HT were significantly higher during SW (*p <* .05).

**Conclusion:**

Hypertension can lead to cognitive impairment in the elderly, including executive function and walking function decline. As a result of these functional declines, elderly patients with hypertension are unable to efficiently allocate brain resources to support more difficult cognitive interference tasks and need to meet more complex task demands by activating more brain regions.

## Graphical abstract

The aim of this study was to explore the differences in the range and extent of cortical activation changes between elderly hypertensive patients and healthy older adults during the performance of a dual cognitive‐walking task, combining cognitive and walking performance, and to identify possible central neural compensatory mechanisms by which the brain responds to cognitive decline in elderly hypertensive patients. This study found that elderly hypertensive patients are unable to allocate brain resources efficiently to support more difficult cognitive interference tasks, and may be able to meet the demands of more complex tasks through greater activation of prefrontal, dorsolateral prefrontal, primary motor cortex, primary somatosensory cortex, and supplementary motor areas. In elderly hypertensive patients, early recognition of cognitive impairment may be possible by means of cognitive‐walking dual‐task brain function tests.

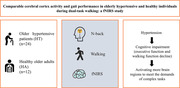

## 1 INTRODUCTION

Compared to healthy individuals, older adults with cardiovascular risk factors (CVRF) such as hypertension usually show poorer cognitive performance, lower physical fitness and altered brain function (Talamonti et al., 2021). Vinyoles et al. (2008) showed that cognitive dysfunction occurs in more than 12% of older hypertensive patients over 60 years of age. Studies (Sanders et al., 2012; Sellers & Newby, 2011) showed that hypertension and pulse pressure in older adults are associated with abnormal balance function, gait and gait speed, and that poor blood pressure control and high pulse pressure significantly reduce gait speed and increase the risk of falls. It is crucial to understand the impact of behavioral and brain function on walking in hypertensive older adults in order to provide targeted interventions (Fraser et al., 2016).

By comparing with single‐task walking, dual‐task walking can better simulate the complex situations faced by older people in daily life, and therefore has higher ecological validity. In older adults, worse dual‐task walking performance was associated with an increased risk of fall, weakness, disability, and death (Goh et al., 2021). Elderly hypertensive patients may face more challenges during walking, such as abnormal blood pressure regulation and atherosclerosis, which may have an important impact on their brain function and gait control. Functional near‐infrared spectroscopy (fNIRS) is a noninvasive neuroimaging technology, which can study the local changes of cerebral blood flow and quantify the changes of oxygen and deoxyhemoglobin concentration related to tasks (Csipo et al., 2019; Tak & Ye, 2014). fNIRS is not sensitive to motion artifacts, is not limited by walking activities, and is portable (Herold et al., 2017; Tak & Ye, 2014). Previous fNIRS studies have been combined with dual tasks to have good applications in cognitive impairment (Cao et al., 2022), stroke (Hermand et al., 2019), Parkinson's disease (Ranchet et al., 2020), diabetes (Holtzer et al., 2018), and other diseases. However, there are few studies on the explanatory factors of the relationship between hypertension and physical function restriction of elderly hypertensive patients by fNIRS, and limited evidence has been shown regarding the effects of dual tasks on cerebral cortex, cognitive performance and gait in hypertensive patients.

fNIRS is a method to assess brain activity by measuring the hemodynamic changes induced by brain activation. Brain activity is associated with various physiological processes that cause changes in the optical properties of brain tissue. Thus, the fNIRS technique measures hemodynamic changes in the cerebral cortex by the “transparency” of brain tissue to near‐infrared (NIR) light. NIR light of appropriate wavelength can be absorbed by blood chromophores or scattered in tissues. The light attenuation is mainly caused by hemoglobin, the main chromophore in the brain. In the brain, oxygenated hemoglobin (HbO) and deoxyhemoglobin (HbR) are usually the major absorbers, which allows optical methods to quantify the major hemodynamic variables. Previous studies have provided evidence of impaired brain autoregulation after high blood pressure. The effect of arterial blood pressure oscillation on Δ[HbO_2_] in hypertensive patients is greater than that in healthy control group (Li and Zhang et al., 2021). This seems to suggest that fluctuations in ABP lead to more transmission of Δ[O2Hb] signals in the brain of hypertensive patients, and that even slight changes in perfusion pressure may lead to changes in cerebral blood flow (Novak et al., 1993). This can be explained by the fact that brain arteries and blood vessel walls become hardened and thickened as a result of the long‐term effects of high blood pressure, which in turn increases the pulsation of blood flow through the arteries of the brain, thus making brain tissue particularly vulnerable to changes in blood pressure. The applicability of the FNIR‐based technique to evaluate the direct interaction between ABP and cerebral hemodynamic oscillations in hypertensive patients was demonstrated.

Previous research has found that the coupling strength of the prefrontal cortex of hypertensive patients in resting and standing states would decrease, which may be due to the changes of the regulatory mechanism of myogenic activities caused by hypertension (Bu et al., 2018). Young and middle‐aged hypertensive patients showed impaired working memory, the combination of fNIRS and cognitive test may provide an important measure of cerebrovascular reserve in patients with essential hypertension (Grant et al., 2015). However, there is still a lack of research focus on cognitive and gait performance of elderly hypertensive patients during dual tasks.

This study aims to explore the changes in gait and brain synergistic function connections in people with and without hypertension under dual‐task walking by functional near‐infrared spectroscopy (fNIRS) and gait analysis equipment to simulate the real situation of walking in the elderly in daily life through walking‐cognitive dual tasks. The objectives of this study were (1) to investigate the difference of cortical activation between the elderly hypertensive patients and the elderly healthy people when performing SC, SW, and DT (2) to explore the difference of gait parameters and working memory between elderly hypertensive patients and elderly healthy people when performing SC, SW, and DT (3) to explore the association between cerebral cortex and gait performance.

## 2 MATERIALS AND METHODS

### 2.1 Participants

This is a cross‐sectional study, referring to previous similar studies (Kvist et al., 2023; Salzman et al., 2021); we used a medium effect size of 0.3 (*f*) with a power of 0.80, alpha level of 0.05, and a correlation between repeated measures of 0.5. The power analysis indicated that a minimum of 20 participants was required. A total of 36 subjects, including 12 healthy older adults (HA) and 24 older hypertensive (HT) patients, were recruited for this study by recruiting participants to the community. The inclusion criteria were as follows: (a) over age 60; (b) the older hypertensive patients were diagnosed with hypertension by their physicians (a diagnosis of hypertension was performed when systolic blood pressure ≥140 mm Hg or diastolic blood pressure ≥90 mm Hg) and took antihypertensive drugs daily; (c) normal vision and hearing; (d) no limb movement disorder and obstacles to walking. The exclusion criteria were as follows: (a) Montreal Cognitive Assessment (MoCA) score < 22 points; (b) limb movement disorders and abnormal gait; (c) with other medical history or diseases that may affect the experimental results, such as stroke, intracranial space occupying lesions, neuropsychiatric disorders, diabetes mellitus, peripheral neuropathy, orthopedic diseases. All subjects signed the informed consent form. The protocol was approved by the Medical Ethics Committee of Zhujiang Hospital, Southern Medical University, Guangzhou, Guangdong, China (2022‐KY‐083‐01).

### 2.2 Clinical evaluation

The cognitive abilities of the subjects were screened by the Montreal Cognitive Assessment (MoCA) scales (Nasreddine et al., 2005). Participates’ fear of falling was assessed by the Activities‐specific Balance Confidence (ABC) Scale (Powell & Myers, 1995) and symptoms of depression were measured by the Center for Epidemiological Studies Depression Scale (CES‐D) (Zhang et al., 2011). After resting for 20 min before the beginning of the experiment, systolic and diastolic blood pressures of the left and right hands of the subjects were measured once each using an electronic sphygmomanometer with an arm cuff (model U10K, Omron, China), and the average value was taken.

### 2.3 fNIRS acquisition and analysis

A portable 35‐channel near‐infrared functional brain imaging device (NirSmart, Danyang Huichuang Medical Equipment Co., Ltd., China) was used to record brain activity. The used wavelengths are 730 and 850 nm. The whole channel sampling rate of the equipment is 10 Hz. The equipment meets the requirements of the internationally used 10/20 electrode distribution system.

We selected to measure subjects’ left anterior prefrontal cortex (L‐APFC), right anterior prefrontal cortex (R‐APFC), left dorsolateral prefrontal cortex (L‐DLPFC), right dorsolateral prefrontal cortex (R‐DLPFC), left somatosensory cortex (L‐S1) and right somatosensory cortex (R‐S1), left primary motor cortex (L‐M1), right primary motor cortex (R‐M1), left supplementary motor area (L‐SMA), right supplementary motor area (R‐SMA), because these regions are related to the functions of cognition and motion (George et al., 2019). We selected the HbO_2_ concentration as a marker of cortical activity because it is the most sensitive and reliable indicator of locomotion‐related changes in regional cerebral oxygenation (Holtzer et al., 2020; Miyai et al., 2001).

The NirSpark software package (HuiChuang, China) was used for analyzing fNIRS data, removing the artifacts unrelated to the experimental data, choosing the band‐pass filter (0.01–0.2 Hz) for the noise and interference signals to filter; optical density was converted to blood oxygen concentration; “5∼30 s” was set as a block paradigm time for hemodynamic response function (HRF) and “0∼30 s” as the reserved baseline state. A general linear model (GLM) was used to analyze the HbO_2_ time series data. The beta value, which reflects the level of cortical activation of the channel, was used as an estimate of the HRF prediction of the HbO_2_ signal and can be used to represent the peak value of the HRF function (Cao et al., 2022).

### 2.4 Quantitative gait assessments

Intelligent Device for Energy Expenditure and Activity (IDEEA^®^, MiniSun LLC) equipment was used to record various parameters of participants including the time, step length, step speeds, step frequency, stride length. The main recorder of IDEEA system was secured on the left waistband. One sub‐recorder was taped above each lateral malleolus. Five sensors were placed on the sternum and bilaterally on the plantar aspect of foot and midline of the anterior aspect of thigh. The equipment has excellent reliability and has been verified in other studies (Li et al., 2021; Maffiuletti et al., 2008).

### 2.5 Cognitive task

The n‐back task is a reliable cognitive task that affects executive working memory and engages multiple areas in the cortex (Doumas et al., 2009). And the 2‐back task was considered a high difficulty level. It requires participants to attend to a string of stimuli (e.g., number) and respond as quickly as possible when a stimulus is presented that is the same as the stimulus two positions prior, and provides the optimal level of cognitive challenge for elderly (Bopp & Verhaeghen, 2020; Fraser et al., 2016).

The numbers (1–9) were played on the computer by an E‐prime 3.0 Psychology Software Tools Incorporated. It presents 20 numbers in a pseudo‐random order, with each stimulus lasting 0.5 s and 1 s apart. Participants were instructed to listen to the string of numbers and press the button (held in their right hand) when they heard the same number as the stimulus two positions prior, they pressed the “Yes” button, otherwise, the “No” button. Before the experiment started, the subjects briefly practiced the 2‐back task until they could correctly respond to at least 70% of the single cognitive task stimuli (Shigeta et al., 2021).

We calculated the 2‐back task accuracy, the rate of missing press, reaction time, and dual‐task cost (DTC) of participants as the outcome indicators. DTC using the following formula: $DTC\left(\%\right)=\frac{Dual-taskperformance-single-taskperformance}{single-taskperformance}\times 100\%$. Larger DTC means worse performance under dual‐task conditions.

### 2.6 Experimental protocol

**FIGURE 1:**
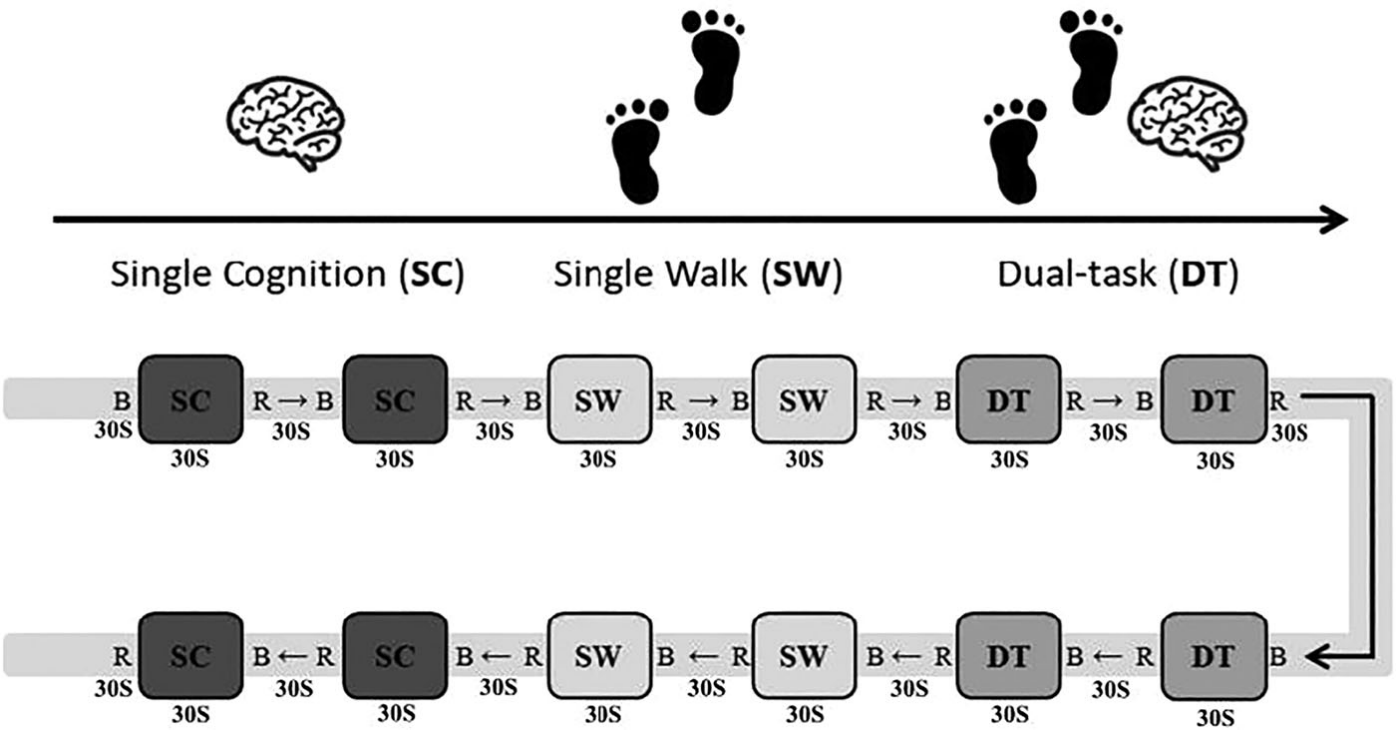
Schematic diagram of the task. SC: single cognitive task, SW: single‐task walk, DT: dual‐task walk, B: baseline, R: rest.

The experimental protocol consists of three conditions: a single cognitive task (SC), a single‐task walk (SW), and a dual‐task walk (DT). The SC required subjects to perform 2‐back task while standing. During the SW, the subjects were asked to walk freely and straightly at their usual speed in a 40 m corridor. During the DT, subjects were asked to complete the 2‐back task while walking and were instructed to pay equal attention to both tasks to minimize task prioritization effects. Each experimental condition was managed in a block of 30 s, and then the subjects were instructed to rest for 30 s. We repeated each task four times, and the complete sequence of the stimulus block was presented according to the A‐B‐B‐A design, as follows: SC‐SC‐SW‐SW‐DT‐DT‐DT‐DT‐SW‐SW‐SC‐SC. This type of design can effectively control the fatigue effect of subjects under various conditions; reliability and validity for this paradigm had been well established (Doumas et al., 2009; Talamonti et al., 2021). The procedure is as shown in Figure 1.

### 2.7 Statistical analyses

Data on the results were checked for normality (Shapiro–Wilk test) and homogeneity of variance (Levene test) to see if parametric analysis assumptions were met. Data were presented as *n* (%) for categorical variables, mean (standard ± deviation) for normally distributed variables, or median (quartiles) for continuous data with nonnormal distribution. When normality was obeyed, a paired *t*‐test was used for intragroup comparisons and two independent samples *t*‐test for between‐group comparisons, otherwise, a nonparametric test was considered. Categorical variables were compared using the chi‐squared test. Pearson's correlation coefficient is used for bivariate normal distribution data, and Spearman's correlation coefficient is used for bivariate nonnormal distribution data. All analyses were performed on SPSS version 23, IBM, and *p *< .05 was considered statistically significant.

## 3 RESULTS

### 3.1 Analysis the demographics characteristics and clinical measurements between HA and HT

A total of 36 subjects were included in this study, 24 hypertensive older adults with a median age of 68.0 ± 3.0 years and 12 healthy older adults with a mean age of 65.0 ± 4.4 years. Activity Balance Confidence Scale scores were higher in the healthy older adults than in the hypertensive older adults (*p* < .05) (Table 1).

**TABLE 1:** Characteristics of HA and HT (M ± SD).

Variable	HA (*N* = 12)	HT (*N* = 24)	*p* Value
Gender (female/all)	8/12	12/24	.481
Age (years)	65.0 ± 4.4	68.0 ± 3.6	.173
Body weight(kg)	61.5 ± 8.7	61.5 ± 7.9	.708
Body height (cm)	158.5 ± 8.3	160.5 ± 7.1	.525
BMI	23.2 ± 3.3	24.2 ± 2.1	.446
Years of education (years)	12.0 ± 2.6	12.0 ± 2.8	.350
Course of hypertension (years)	/	6.5 ± 9.1	/
Stages of hypertension			
Stage 1	/	24	/
Stage 2	/	0	/
Stage 3	/	0	/
Resting blood pressure (mmHg)			
Systolic pressure	121.5 ± 15.9	135.5 ± 14.4	.001
Diastolic pressure	76.0 ± 5.4	85.5 ± 9.0	.006
Fall in the last 12 months			1.000
	Yes = 2	Yes = 4	
	No = 10	No = 20	
MoCA (max = 30)	27.0 ± 1.7	27.0 ± 1.9	.625
ABC (max = 100)	96.9 ± 1.9	91.8 ± 8.3	<.001
CES‐D (max = 60)	3.0 ± 3.2	3.5 ± 4.4	.685
DTC (%)	−15.0 ± 12.9	−5.3 ± 18.9	.127

BMI: body mass index, MoCA: Montreal Cognitive Assessment Scales, ABC: Activities‐specific Balance Confidence Scale, CES‐D: Center for Epidemiological Studies Depression Scale, DTC: dual‐task cost.

### 3.2 Analysis the activation of HbO_2_ across groups during SW, SC and DT

**FIGURE 2:**
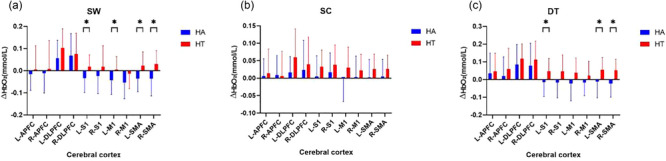
Analysis the activation of changes in HbO_2_ concentration between groups during SW, SC, and DT. *p < .05. (a) Analysis of HbO_2_ concentration difference among groups during SW. (b) Analysis of HbO_2_ concentration difference across groups during SC. (c) Analysis of HbO_2_ concentration difference across groups during DT, HA: healthy older adults group, HT: older hypertensive group, SW: single‐task walk, SC: single cognitive task, DT: dual‐task walk, L‐APFC: left anterior prefrontal cortex, R‐APFC: right anterior prefrontal cortex, L‐DLPFC: left dorsolateral prefrontal cortex, R‐DLPFC: right dorsolateral prefrontal cortex, L‐S1: left somatosensory cortex, R‐S1: right somatosensory cortex, L‐M1: left primary motor cortex, R‐M1: right primary motor cortex, L‐SMA: left supplementary motor area, R‐SMA: right supplementary motor area.

During the SW task, the cerebral cortex activation of L‐S1, L‐M1, L‐SMA, and R‐SMA of HA was significantly lower than HT (*p *< .05). During the DT task, the cerebral cortex activation of L‐S1, L‐SMA, and R‐SMA of HA was significantly lower than HT (*p *< .05), but the activation of other cerebral cortex was no significant difference across groups (*p *> .05). There was no significant difference during SC task across groups (*p *> .05), as shown in Figure 2.

### 3.3 Intragroup analysis of HbO_2_ results

**FIGURE 3:**
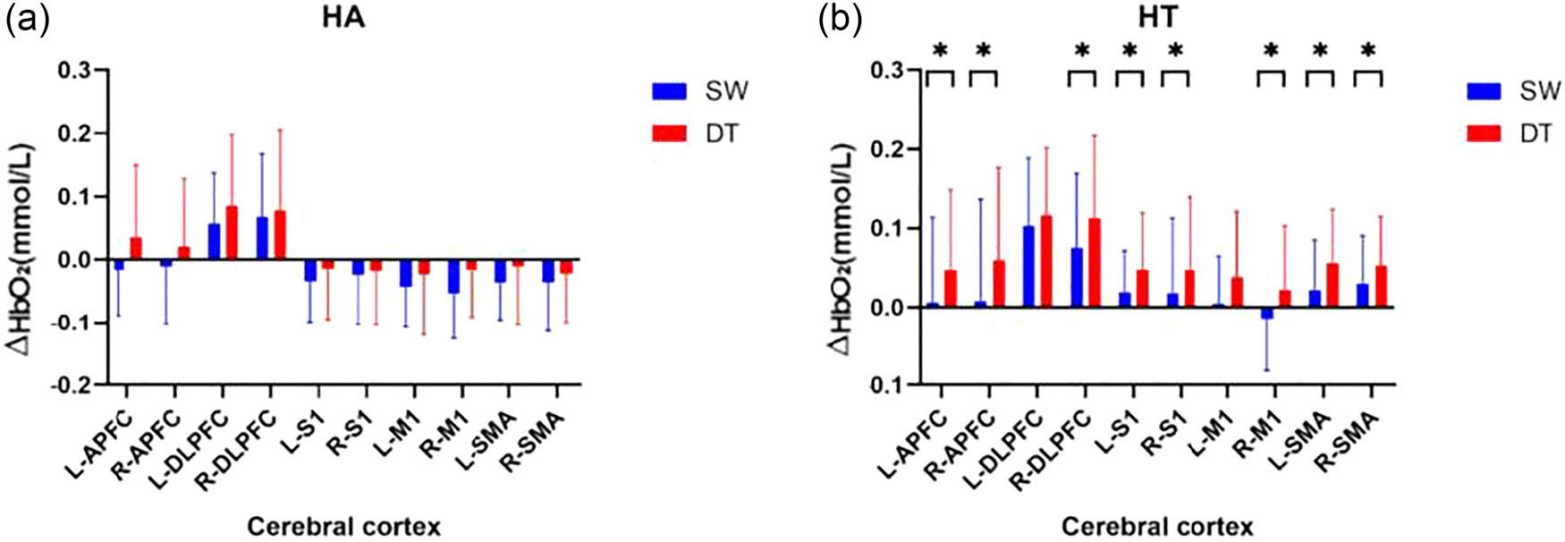
Analysis of HbO_2_ concentration difference results intragroup during SW and DT. *p < .05. (a) Analysis of HbO_2_ concentration difference within HA group during SW and DT. (b) Analysis of HbO_2_ concentration difference within HT group during SW and DT, HA: healthy older adults group, HT: older hypertensive group, SW: single‐task walk, DT: dual‐task walk, L‐APFC: left anterior prefrontal cortex, R‐APFC: right anterior prefrontal cortex, L‐DLPFC: left dorsolateral prefrontal cortex, R‐DLPFC: right dorsolateral prefrontal cortex, L‐S1: left somatosensory cortex, R‐S1: right somatosensory cortex, L‐M1:left primary motor cortex, R‐M1: right primary motor cortex, L‐SMA: left supplementary motor area, R‐SMA: right supplementary motor area.

**FIGURE 4:**
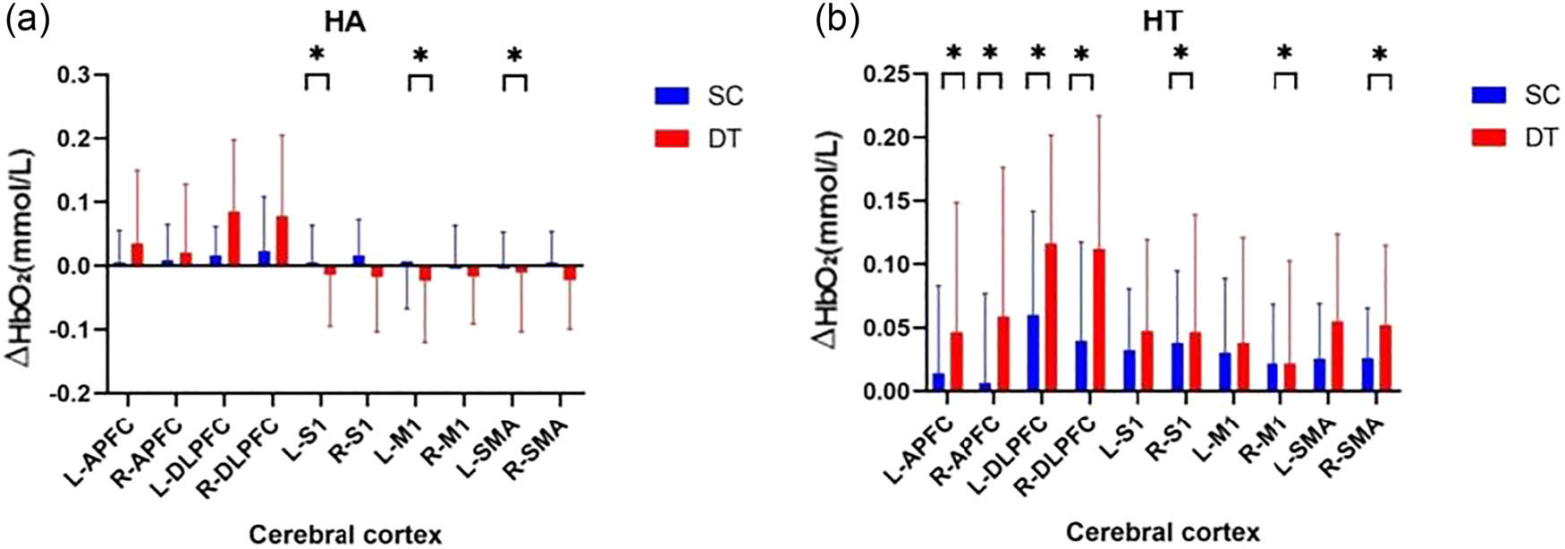
Analysis of HbO_2_ concentration difference intragroup during SC and DT *p < .05. (a) Analysis of HbO_2_ concentration difference within HA group during SC and DT. (b) Analysis of HbO_2_ concentration difference within HT group during SC and DT, HA: healthy older adults group, HT: older hypertensive group, SW: single‐task walk, DT: dual‐task walk, L‐APFC: left anterior prefrontal cortex, R‐APFC: right anterior prefrontal cortex, L‐DLPFC: left dorsolateral prefrontal cortex, R‐DLPFC: right dorsolateral prefrontal cortex, L‐S1: left somatosensory cortex, R‐S1: right somatosensory cortex, L‐M1:left primary motor cortex, R‐M1: right primary motor cortex, L‐SMA: left supplementary motor area, R‐SMA: right supplementary motor area.

Between SW and DT, paired *t*‐test showed that the activation of all measured cerebral cortex in within HA group was no significant difference (*p *> .05). The activation of L‐APFC (*p *< .05), R‐APFC (*p *< .05), R‐DLPFC (*p *< .05), L‐S1 (*p *< .05), R‐S1 (*p *< .05), R‐M1 (*p *< .05), L‐SMA (*p *< .05), and R‐SMA (*p *< .05) during DT is higher than SW within HT group, but the activation of L‐DLPFC and L‐M1 was no significant difference (*p *> .05), as shown in Figure 3. Between SC and DT, paired *t*‐test showed that the activation of L‐S1, L‐M1, and L‐SMA during DT is lower than SC in within HA group (*p *< .05). The activation of L‐APFC (*p *< .05), R‐APFC (*p *< .05), L‐DLPFC (*p *< .05), R‐DLPFC (*p *< .05), R‐S1 (*p *< .05), R‐M1 (*p *< .05), and R‐SMA (*p *< .05) during DT is higher than SC in within HT group, but the activation of L‐S1, L‐M1, and L‐SMA was no significant difference (*p *> .05), as shown in Figure 4.

### 3.4 Analysis of 2‐back task accuracy, the rate of missing press, reaction time and gait parameters of across groups

Two independent samples *t*‐test showed that there was significant difference between the HA and HT groups in accuracy, missing press rate and reaction time during SC (*p *< .05). But there was no significant in accuracy, missing press, and reaction time (*p *> .05) between HA and HT groups during DT. And there was no significant difference in gait parameters between HA and HT groups during SW and DT (*p *> .05), as shown in Table 2.

**TABLE 2:** Analysis of 2‐back task performance and gait parameters of across groups (mean ± SD).

Variable	HA (*N* = 12)			HT (*N* = 24)		
	SW	SC	DT	SW	SC	DT
Accuracy (%)	/	72.34 ± 14.34^a^	60.99 ± 7.19	/	57.58 ± 18.72^a^	53.64 ± 15.87
Missing press (%)	/	8.56 ± 6.80^a^	15.39 ± 6.30	/	26.68 ± 22.27^a^	27.84 ± 19.61
Reaction time (ms)	/	989.07 ± 67.83^a^	998.66 ± 148.14	/	1101.10 ± 151.17^a^	1086.86 ± 126.47
Step speed (m/s)	1.17 ± 0.15	/	1.05 ± 0.22	1.14 ± 0.15	/	1.09 ± 0.19
Step length (m)	0.62 ± 0.08	/	0.57 ± 0.08	0.61 ± 0.05	/	0.59 ± 0.06
Step frequency (steps/min)	111.75 ± 10.12	/	106.60 ± 14.60	111.61 ± 8.45	/	110.27 ± 9.65
Stride length (m)	1.15 ± 0.16	/	1.11 ± 0.17	1.17 ± 0.09	/	1.14 ± 0.11

^a^ *P *< 0.05.

HA: healthy older adults group, HT: older hypertensive group, SC: single cognitive task, SW: single‐task walk, DT: dual‐task walk.

### 3.5 Analysis of 2‐back task accuracy, the rate of missing press, reaction time, and gait parameters of intragroup comparisons

Paired *t*‐test showed that the accuracy in SC was significantly higher than DT (*p *< .05), the missing press rate in SC was significantly lower than DT (*p *< .05), but the reaction time was no significant difference during SC and DT in HA group (*p *> .05). The step speeds, step length, and step frequency in SW were greater than DT in HA group (*p *< .05), but the stride length was no significant in HA group between SW and DT (*p *> .05). There was no significant in accuracy, missing press rate, and reaction time in HT group between SC and DT (*p *> .05). And the step speeds, step length, and stride length in SW were significantly greater than DT (*p *< .05), but the step frequency was no significant in HT group between SW and DT (*p *> .05) (Table 3).

**TABLE 3:** Analysis of 2‐back task performance and gait parameters of intragroup comparisons (mean ± SD).

Variable	HA (*N* = 12)			*p* Value	HT (*N* = 24)			*p* Value
	SW	SC	DT		SW	SC	DT	
Accuracy (%)	/	72.34 ± 14.34	60.99 ± 7.19	.002*	/	57.58 ± 18.72	53.64 ± 15.87	0.078
Missing press (%)	/	8.56 ± 6.80	15.39 ± 6.30	.011*	/	26.68 ± 22.27	27.84 ± 19.61	0.529
Reaction time (ms)	/	989.07 ± 67.83	998.66 ± 148.14	.769	/	1101.10 ± 151.17	1086.86 ± 126.47	0.302
Step speed (m/s)	1.17 ± 0.15	/	1.05 ± 0.22	.013*	1.14 ± 0.15	/	1.09 ± 0.19	0.014*
Step length (m)	0.62 ± 0.08	/	0.57 ± 0.08	.021*	0.61 ± 0.05	/	0.59 ± 0.06	0.001*
Step frequency (steps/min)	111.75 ± 10.12	/	106.60 ± 14.60	.019*	111.61 ± 8.45	/	110.27 ± 9.65	0.239
Stride length (m)	1.15 ± 0.16	/	1.11 ± 0.17	.099	1.17 ± 0.09	/	1.14 ± 0.11	0.038*

* *p *< .05.

HA: healthy older adults group, HT: older hypertensive group, SC: single cognitive task, SW: single‐task walk, DT: dual‐task walk.

### 3.6 Analysis of correlation between HbO_2_ and gait parameters

**FIGURE 5:**
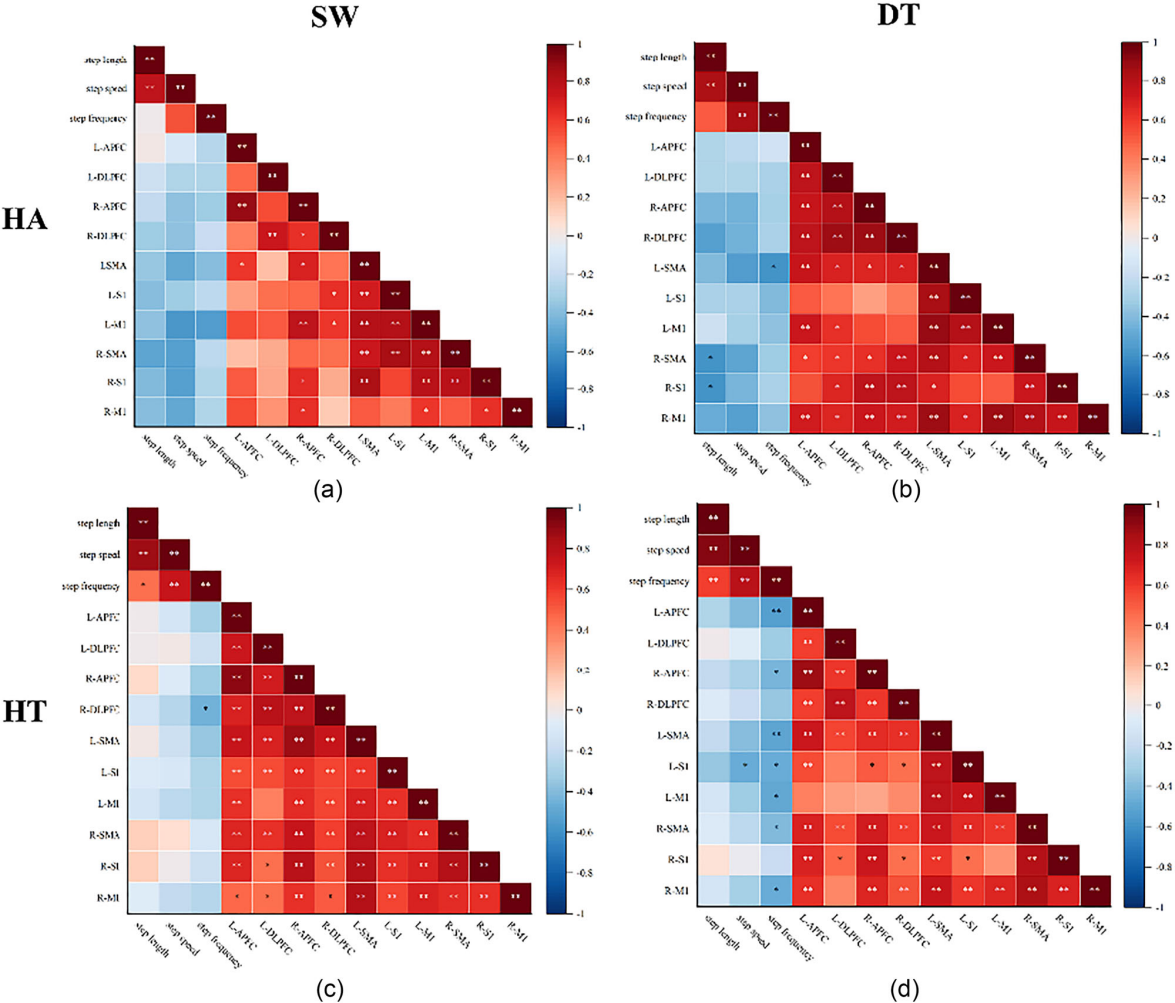
Correlation heat map between cortical activation and gait parameters between the healthy elderly group and the hypertensive elderly group during single‐task walk (SW) and dual‐task walk (DT). HA: healthy older adults group, HT: older hypertensive group, L‐APFC: left anterior prefrontal cortex, R‐APFC: right anterior prefrontal cortex, L‐DLPFC: left dorsolateral prefrontal cortex, R‐DLPFC: right dorsolateral prefrontal cortex, L‐S1: left somatosensory cortex, R‐S1: right somatosensory cortex, L‐M1: left primary motor cortex, R‐M1: right primary motor cortex, L‐SMA: left supplementary motor area, R‐SMA: right supplementary motor area. *p < .05, **p < .01.

We used correlation analysis to show the correlation between HbO_2_ and gait parameters both HA group and HT group. In the HA group, as shown in Figure 5a, during SW, there was no significant correlation between measured cerebral cortex and gait parameters (*p *> .05). During DT, there is a negative correlation between L‐SMA and step frequency (*r *= −0.585, *p *= .046), R‐SMA and step length (*r *= −0.611, *p *= .035), and R‐S1 and step length (*r *= −0.603, *p *= .038). There was no correlation between other measured cerebral cortex and gait parameters in HA group during DT (Figure 5b).

In the HT group, as shown in Figure 5c, during SW, participants’ R‐DLPFC and step frequency (*r *= −0.458, *p *= .024) existed the correlation. There was no correlation between other measured cerebral cortex and gait parameters in the HT group during SW. The results of Figure 5d indicate that during DT, a negative correlation exists between L‐APFC and step frequency (*r *= −0.533, *p *= .007), R‐APFC and step frequency (*r *= −0.437, *p *= .033), L‐SMA and step frequency (*r *= −0.516, *p *= .01), L‐S1 and step speed (*r *= −0.478, *p *= .018), L‐S1 and step frequency (*r *= −0.480, *p *= .018), L‐M1 and step frequency (*r *= −0.493, *p *= .014), R‐SMA and step frequency (*r *= −0.411, *p *= .046), and R‐M1 and step frequency (*r *= −0.467, *p *= .022). There was no correlation between other measured cerebral cortex and gait parameters in HT group during DT.

## 4 DISCUSSION

This article mainly studied the activation of the cortex and its cognitive and walking performance differences between hypertensive elderly and healthy elderly people performing SC, SW and DT. We also explored the association between cerebral cortex and gait performance. These findings are discussed in detail below.

First of all, due to the changes in brain structure and function caused by aging, the elderly have limited attention resources compared to young people, which may lead to changes in executive function and gait (St George et al., 2022). Many previous studies have shown that hypertension can further lead to central structural and functional abnormalities, resulting in PFC damage and neurovascular disorders through a variety of pathophysiological mechanisms (Bu et al., 2018; Canavan & O'Donnell, 2022). Due to the long‐term effects of high blood pressure, the brain arteries and blood vessel walls harden and thicken, making brain tissue particularly vulnerable to changes in blood pressure, and brain activation is more pronounced than in healthy controls. This can lead to a further decline in attention resources in older adults and further deterioration in executive function and gait. In this study, there were no significant differences in cognitive and walking performance in HT when performing dual tasks compared with HA, but they showed higher cortical activation in L‐M1 and bilateral SMA, indicating that elderly hypertensive patients need to recruit more central resources for functional compensation. Such results may be similar to the previous theory of neural inefficiency, where higher brain activation is associated with equivalent or poor task performance, suggesting that the brain does not allocate resources efficiently to support task demands (Holtzer et al., 2018; Rypma et al., 2002).

The increased variability of blood pressure in older adults may also increase their risk of global cognitive, executive, and language impairment. In a previous study, meeting physical activity guidelines was associated with higher cognitive function in a national sample of hypertensive older adults. With the increase of risk level, the systolic blood pressure, pulse pressure, and disease duration of the elderly with hypertension gradually increased, which may lead to the gradual decline of their performance in cognitive function tests. The elderly with hypertension have a better cardiorespiratory fitness status with a higher level of physical activity. Physical activity can have a beneficial effect on cognitive function by improving cardiorespiratory fitness in the elderly with hypertension (Frith & Loprinzi, 2017).

Since PFC is a key region of the cerebral cortex that controls executive function and walking in older adults, it is especially prominent in dual‐task conditions that combine cognition and motor (Beauchet et al., 2016). Previous studies of cognitive interference dual tasks in the elderly have mostly found changes in PFC activation between groups when performing more complex tasks, but for the elderly with hypertension in this study, when performing the DT, no increase or decrease in PFC compensatory activation was observed compared with the healthy group, and the compensatory activation was concentrated in the M1 and SMA. This finding in this study is different from most previous studies. In response to this condition, the reasons that may lead to this result are analyzed as follows: first of all, due to the limited number of elderly hypertensive patients included in this study, 24 of the hypertensive subjects were all graded with hypertension grade 1, which may lead to less impact of hypertension on individuals, and compared with hypertensive elderly people with higher cognitive impairment, it is possible that the structure and function of PFC in hypertensive elderly people in this study are relatively complete, so that they have more cognitive resources to compensate for the dual‐task paradigm (Buckner, 2004; Cabeza et al., 2002). Second, some studies suggest that a sudden drop in blood pressure and orthostatic hypotension may contribute to an increased risk of falls, depending on the type, dose, and duration of antihypertensive medications (Shen et al., 2015; Tinetti et al., 2014). It is also believed that regular antihypertensive therapy with β blockers in hypertensive patients can reduce the risk of cognitive impairment, and the degree of cognitive impairment in hypertensive patients with stable blood pressure control is significantly lower than that in patients with uncontrolled hypertension (Gelber et al., 2013). However, studies do not support the effect of antihypertensive drugs in improving cognitive function in hypertensive patients (Peters et al., 2014). The effect of antihypertensive drugs on cognitive function in hypertensive patients is controversial. All participants in the geriatric hypertension group in this study were given antihypertensive drugs for a certain period of time, and because the type and duration of antihypertensive drugs were not taken into account in this study, it was not possible to know the potential effect of antihypertensive drugs on cognitive function and other outcomes in older hypertensive subjects. In addition to this, older adults with hypertension may have a “posture‐first strategy” when performing cognitive‐walking dual tasks (Holtzer et al., 2016).

The 2‐back working memory test of subjects in this study found that the cognitive performance of single cognitive tasks in elderly hypertensive patients was significantly poor, but no significant differences were observed in the activation levels of various brain regions, including PFCs. Combined with cognitive scale, working memory task performance, and cortical activation, it can be found that although elderly hypertensive patients have normal performance on cognitive scale scores, and no changes in cortical activation in elderly hypertensive patients in coping with simple working memory tasks have been observed, they have significant decline in working memory function when coping with working memory tasks, which may reflect the decline of executive function in elderly hypertensive patients to a certain extent. Studies have shown that cognitive tasks combined with cognitive inhibition interfere with walking more than tasks requiring working memory alone (St George et al., 2022). Therefore, it is also possible to combine dual tasks in older hypertensive patients who may have cognitive impairment to examine different cognitive tasks to further clarify whether they have cognitive decline.

From a clinical point of view, older people with comorbid hypertensive disease are at higher risk of developing cognitive impairment, and they may already have manifestations of cognitive impairment to varying degrees, which will affect their activities of daily living and may lead to executive function decline, falls, and mobility impairment. Therefore, it is valuable to assess the cognitive performance and gait parameters of elderly patients with hypertension through cognitive‐walking dual tasks. In addition to this, designing dual‐task interventions in older adults with hypertension may help reduce cognitive decline and delay the onset and progression of cognitive decline before it occurs.

There were some limitations to this study. First, the age of the subjects in this study ranged from sixty to seventy years, and cortical activation in elderly hypertensive patients over seventy years of age and beyond was unknown. As well, the elderly hypertensive subjects recruited for this study had a low severity of hypertension, which was limited to hypertension grade 1, and it was not possible to determine the effect of more severe hypertension on altered cortical activation, and future studies should recruit a wider range of age groups as well as elderly subjects in different hypertension grades. Second, this study did not collect and analyze drug‐related data such as the type, dose, and duration of antihypertensive drugs in elderly patients with hypertension, ignoring the possible impact of antihypertensive drugs on the results. In addition, the sample size of this study was only 36 people, which may have been selected for bias. Despite the relatively small sample size and the uneven size of the control and experimental groups, this study yielded meaningful results, but further large studies are needed to validate our findings.

## 5 CONCLUSION

Elderly patients with hypertension cannot effectively allocate brain resources to support more difficult cognitive interference tasks and may meet more complex task requirements by activating more APFC, DLPFC, M1, S1, and SMA. For elderly patients with hypertension, it is particularly important to carry out comprehensive examination of cognitive function and gait before they develop cognitive impairment, and it is particularly important to further combine fNIRS with cognitive‐walking dual‐task cortical activation status, cognitive paradigm, and gait parameters for elderly hypertensive patients with normal cognitive scales or no obvious abnormalities in gait parameters, in order to identify and prevent hypertensive patients with cognitive impairment in clinical early stage.

## AUTHOR CONTRIBUTIONS

**Qiuru Yao**: Writing—original draft; writing—review and editing. **Ling Chen**: Writing—original draft; writing—review and editing. **Hang Qu**: Data curation; investigation. **Weichao Fan**: Software; investigation. **Longlong**: Writing—review and editing. **Gege Li**: Investigation. **Jinjing Hu**: Investigation. **Jihua Zou**: Project administration; resources. **Guozhi Huang**: Conceptualization; supervision. **Qing Zeng**: Project administration; conceptualization.

## CONFLICT OF INTEREST STATEMENT

The authors declare that the research was conducted in the absence of any commercial or financial relationships that could be construed as a potential conflict of interest.

### PEER REVIEW

The peer review history for this article is available at https://publons.com/publon/10.1002/brb3.3568.

## Data Availability

The raw data supporting the conclusions of this article will be made available by the corresponding author on reasonable request.
